# EEG imagined speech neuro-signal preprocessing and deep learning classification

**DOI:** 10.1038/s41598-026-39395-6

**Published:** 2026-03-30

**Authors:** Fatma Elwasify, Eman Shaaban, Randa M. Abdelmoneem

**Affiliations:** Computer Systems Department, Faculty of Computer and Information Science, Ain Shams University, Cairo, Egypt

**Keywords:** Imagined speech, EEG, Brain-computer interface, Deep learning, CNN-LSTM, LOSO cross-validation, ICA, Frequency-domain filtering, Cross-subject generalization, Software, Learning algorithms, Network models, Neural decoding, Machine learning

## Abstract

This study presents an advanced approach for classifying imagined speech from Electroencephalography (EEG) signals, leveraging deep learning architectures and tailored preprocessing techniques. Five Convolutional Neural Network (CNN)–Long Short-Term Memory (LSTM) hybrid architectures are proposed and investigated, to extract spatial and temporal features in EEG signals, in conjunction with a proposed six-phase preprocessing pipeline combining Independent Component Analysis (ICA) for artifact attenuation with zero-phase Frequency-Domain Filtering (FD-F) and adaptive normalization. The proposed approach is evaluated across single- and multi-category classification and across multiple cross-validation strategies including random splits, GroupKFold and Leave-One-Subject-Out (LOSO) using weighted metrics, per-class, and per-subject analysis. Experiment results demonstrate the superior performance achieved by FD-F, and that by integrating the most effective proposed bidirectional temporal modeling architecture CNN-2-Bi-LSTM, with the proposed preprocessing pipeline, the approach achieves higher accuracy (exceeding 99%) for 30-class classification maintaining cross-subject generalization against state-of-the-art.

## Introduction

Brain–Computer Interfaces (BCIs) enable direct communication between the human brain and external systems without reliance on muscular activity, offering a transformative communication pathway for individuals with severe motor impairments such as amyotrophic lateral sclerosis (ALS), locked-in syndrome, and spinal cord injury^1–3^. Among existing BCI paradigms, imagined speech represents one of the most intuitive and natural modalities, as it seeks to decode neural activity associated with internally articulated words without overt vocalization^4–6^. In contrast to motor imagery-based BCIs, which require users to perform abstract mental tasks, imagined speech directly exploits the brain’s intrinsic speech production mechanisms, thereby enabling more natural, expressive, and scalable communication^7,8^. In addition, it can be utilized in speech reconstruction through mapping EEG signals to audible speech, opening avenues for direct thought-to-speech systems^9^.

Electroencephalography (EEG) provides the most practical neuroimaging modality for imagined speech BCIs due to its non-invasiveness, portability, low cost, and high temporal resolution^10,11^. However, EEG-based imagined speech classification confronts three fundamental challenges limiting real-world deployment. First, severe signal contamination from physiological artifacts—including electrooculographic (EOG) artifacts from eye movements and blinks, electromyographic (EMG) artifacts from facial muscle tension, and cardiac interference substantially degrades signal quality. Preprocessing techniques, such as bandpass filtering and artifact removal can drastically improve classification accuracy by focusing on the most relevant signal components^12–14^. Second, poor cross-subject generalization arises from individual variability in brain anatomy, electrode positioning, and cognitive strategies, resulting in substantial inter-subject differences in EEG patterns^15,16^. Third, limited vocabulary size constrains practical applicability, as previous work predominantly addresses small vocabularies (≤ 10 classes) with limited exploration of larger vocabularies necessary for functional communication^17–19^. Scaling to 30 classes introduces increased class confusion, reduced discriminability, and higher computational complexity.

Recent advances in deep learning have demonstrated promise for EEG-based BCI applications. Convolutional Neural Networks (CNNs) effectively extract spatial patterns from multi-channel EEG, while Recurrent Neural Networks (RNNs), particularly Long Short-Term Memory (LSTM) networks, capture temporal dependencies in sequential brain signals. Hybrid CNN-LSTM architecture combines these complementary strengths, with CNNs learning spatial filters and LSTMs modeling temporal dynamics^20–22^. More recently, transformer-based architectures have been applied to imagined speech classification, demonstrating the potential of attention mechanisms for EEG analysis^23,24^. However, critical gaps persist. Existing preprocessing approaches rely on simple temporal filtering (bandreject 4–15 Hz^18^, bandpass of 40 Hz^25^ without systematic artifact removal, leaving substantial noise contamination. Validation methodologies employing random split or k-fold cross-validation without subject separation overestimate performance due to data leakage, with limited systematic analysis of cross-subject generalization. While recent architectures show improved performance, they often impose prohibitive computational costs unsuitable for real-time BCI, and systematic comparison of LSTM architectural variants (unidirectional versus bidirectional, varying depth) remains incomplete.

This work addresses these limitations through a comprehensive approach combining architectural impact, advanced preprocessing, and rigorous validation. The main contributions of this research are:

1. *Evaluation of deep learning architectures for imagined speech EEG*: Five CNN–LSTM–based architectures are proposed adopting and adapting^18^ for imagined speech classification on the Kumar dataset^26^, including CNN-1-LSTM, CNN-2-LSTM, CNN-2-Bi-LSTM, CNN-3-LSTM, and 3-LSTM. These architectures are evaluated across single- and multi-category classifications, demonstrating improved scalability to larger imagined speech vocabularies.
2. *Frequency-domain preprocessing for improved EEG signal quality*: An ICA-Assisted Frequency-Domain Filtering (FD-F) signal preprocessing pipeline is introduced, a six-phase pipeline combining Independent Component Analysis (ICA) for artifact attenuation with zero-phase frequency-domain filtering and adaptive normalization. Systematic comparisons indicate that this pipeline provides consistent performance improvements over commonly used temporal filtering approaches.
3. *Rigorous validation strategies*: Models evaluation is conducted using multiple cross-validation strategies including *random splits*, *GroupKFold* cross-validation to mitigate temporal leakage, and *Leave-One-Subject-Out (LOSO)* evaluation with a limited calibration phase, enabling a realistic assessment of cross-subject generalization.
4. *Large-vocabulary imagined speech classification*: By integrating the most effective architecture with the proposed preprocessing pipeline, the proposed approach achieves higher accuracy than previously reported results under comparable validation settings (exceeding 99%) under random-split validation for the full 30-class imagined speech vocabulary of the Kumar dataset, providing an upper-bound performance reference. Using LOSO with calibration, the proposed approach maintains substantially improved cross-subject performance compared to baseline methods, demonstrating its practical applicability to large-vocabulary imagined speech BCIs.

The remainder of this paper is organized as follows: Sect.  2 reviews related work in imagined speech BCIs and deep learning architectures. Section  3 describes the methodology including dataset, preprocessing pipeline, proposed architectures, and validation strategies. Section  4 presents comprehensive results demonstrating contributions from preprocessing, architectures, and their integration, followed by rigorous cross-subject evaluation. Section  5 concludes with key contributions and impact.

## Related work

The field of EEG-based imagined speech recognition has evolved through several methodological paradigms, transitioning from foundational signal processing and classical machine learning to complex deep learning frameworks. Early research prioritized hand-crafted feature engineering and statistical signal decomposition to handle the non-stationary nature of EEG data. However, as vocabularies have scaled, the focus has shifted toward automated feature extraction. Current literature encompasses a broad spectrum of approaches, including pure signal processing for noise attenuation, traditional machine learning (ML) for interpretable classification, and diverse deep learning (DL) architectures—ranging from Convolutional and Recurrent Neural Networks (CNNs/RNNs) to Transformers and Generative Adversarial Networks (GANs)—to address the spatial-temporal complexities of neural speech articulation^11,14,19,27–32^.The effectiveness of analyzing physiological signals in the time-frequency domain has been well-documented in recent literature. For instance, Torghabeh et al.^33^. utilized wavelet coherence analysis to capture intricate interactions between gait signals, transforming these representations into color-coded images for deep transfer learning classification. Their study highlights the critical role of temporal windowing, demonstrating that 10-second segments yielded significantly higher accuracy (99.20%) compared to shorter intervals for four-class task.

García-Salinas et al.^25^ created an electroencephalograms (EEG) dataset comprising five Spanish words (“up,” “down,” “left,” “right,” “select”). The approach utilized Bag of Features (BoF) for signal representation, K-means clustering for feature generation. They proposed Naive Bayes classifier, and transfer learning for imagined speech classification. The study achieved an accuracy of 65.65% ± 13.39 using Naive Bayes classifier and accuracy reduction to 58.74 ± 13.39% for “up” and 61.38 ± 12.47% for “down” using transfer learning.

Kamble et al.^17^ investigated the feasibility and performance of spectral features of EEG signals for imagined speech recognition. The study divided EEG signals into six frequency bands and transformed them into time-frequency representation (TFR) images. A Convolutional Neural Network (CNN) is then used to extract features from the TFR images and classify them into binary and multi-class categories of imagined speech. The evaluation is conducted using EEG-based Imagined Speech Dataset of 15 imagined words (“help,” “light,” “pain,” “stop,” “yes,” “no,” “right,” “left,” “thank you,” “backward,” “down,” “toilet,” “television,” “water,” “medicine”) achieving 51.44% ± 3.55% accuracy. The study specifically utilized the Leave-One-Subject-Out (LOSO) validation scheme to test the cross-subject generalization of their model, achieving 50.42% ± 2.18% accuracy for the multi-class classification for 15-class task.

Abdulghani et al.^34^ proposed a method for classifying imagined speech using EEG signals and deep learning techniques. The researchers utilized an 8-channel EEG headset to collect data from four healthy subjects imagining the commands “up,” “down,” “left,” and “right.” They applied wavelet scattering transformation for feature extraction and employed LSTM-RNN architecture for classification. The model achieved an overall accuracy of 92.50%, demonstrating the potential of deep learning in developing brain-computer interfaces (BCIs) for assisting paralyzed patients.

Alharbi and Alotaibi^35^ presented a framework for classifying imagined speech using EEG data. The authors transformed EEG signals into sequential topographic brain maps and applied hybrid deep learning models, combining 3D-CNN and RNNs. The researchers utilized the BCI2020 dataset^36^, working on 5 imagined words “Hello,” “Help me,” “Stop,” “Thank you,” and “Yes.” The study achieved an average accuracy of 77.8% for word-pair classification and 44.7% for multi-word classification.

The integration of advanced preprocessing with hybrid deep learning models is a key trend for enhancing multi-class detection tasks. Modaresnia et al.^37^ demonstrated that specific image enhancement techniques, such as CLAHE, are foundational to the performance of convolutional neural networks (CNNs) in multi-level classification tasks. Furthermore, they illustrated the efficacy of using a Genetic Algorithm (GA) to tune non-trainable hyperparameters within a modified CNN framework, achieving an accuracy of 99.81% for four-class classification.

The Kumar Imagined Speech EEG Dataset^26^ is a widely used benchmark for imagined speech recognition that has been utilized in various studies to evaluate the effectiveness of different approaches, owing to its diverse range of classes and categories. At first, Kumar et al.^26^ created the Kumar dataset consisting of three categories of characters, digits, and objects with 10 classes each and implemented traditional machine learning models, such as Random Forest achieving moderate accuracy of 66.9%, 68.5%, and 65.7% for the characters, digits, and objects subsets, respectively.

Following that, more researchers have used Kumar benchmark to evaluate their proposed deep learning architectures^18,24,38^.

Tripathi^18^, a hybrid CNN combined with LSTM model is employed to leverage the strengths of both convolutional layers for spatial feature extraction and LSTM layers for temporal feature learning. This method demonstrated significant improvement, with accuracies of 87.3%, 85.9%, and 87.5% for the characters, digits, and objects subsets, respectively.

Ignazio et al.^24^ utilized CNN combined with Transformers to classify imagined speech achieving accuracies of 97.3%, 97.2%, and 96.6% for the characters, digits, and objects subsets, respectively.

Tirupattur et al.^38^, introduces a novel framework to decode and visualize human thoughts using EEG signals. By leveraging a conditional Generative Adversarial Network (GAN), the method transforms EEG signals, captured during thought processes involving digits, characters, or objects, into visual representations. Experimental results show and achieve of 5.439 inception score for the objects category.

These studies demonstrate the progression of methods applied to different EEG imagined speech datasets, highlighting the increasing effectiveness of deep learning architectures in imagined speech classification.

## Methodology

**Fig. 1:**
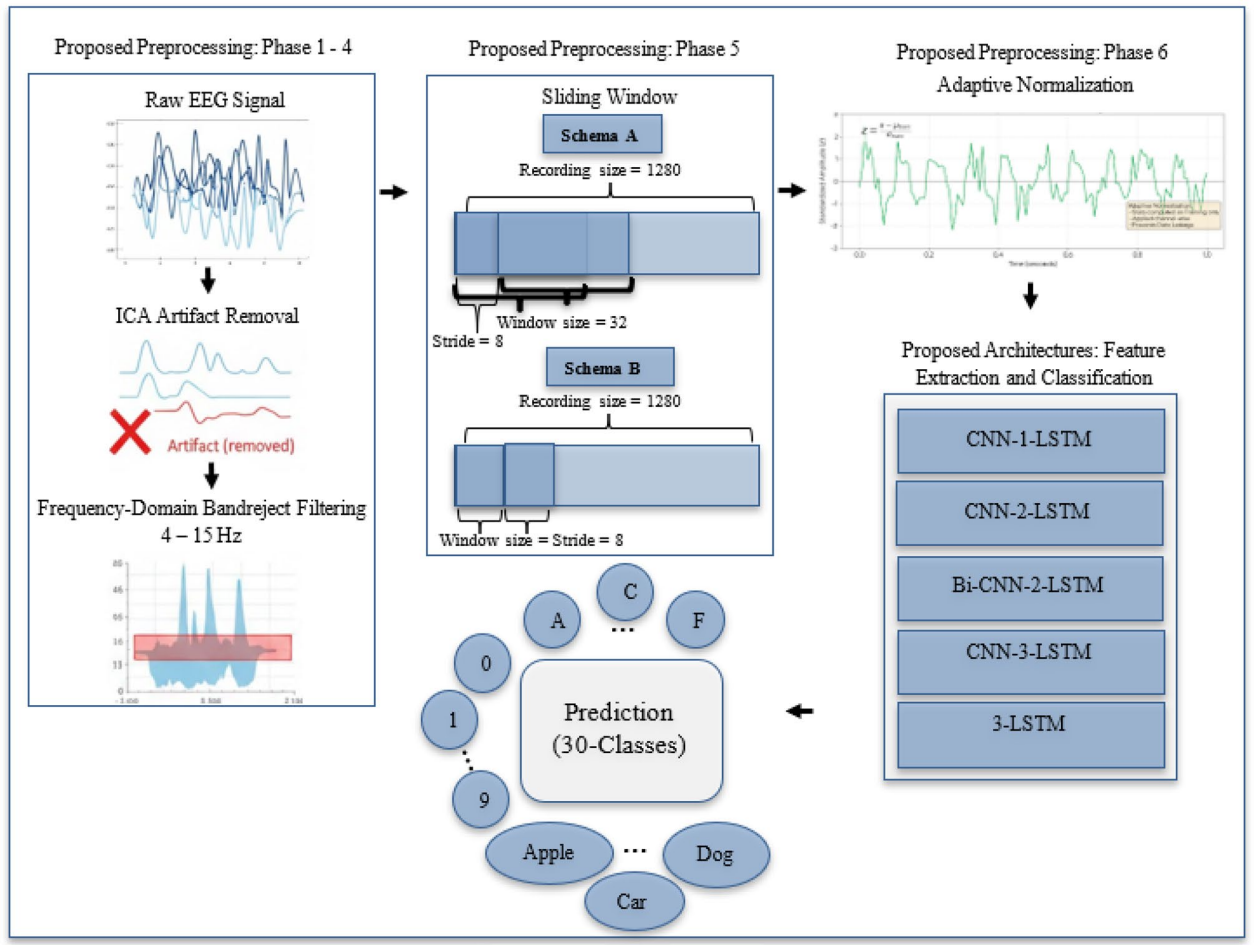
High level diagram of proposed approach that consists of the proposed preprocessing pipeline and the proposed architectures.

In this section, the selected dataset, proposed preprocessing methods, proposed deep learning architectures, experimental setup, cross-validation strategies, and evaluation metrics are described. High level diagram for the proposed approach is shown in Fig. 1 and its detailed description is provided on the following subsections.

### Dataset

Kumar Imagined Speech EEG Dataset is used^26^, a publicly available benchmark dataset containing raw EEG signals from participants imagining 30 classes grouped into three categories: 10 English alphabet characters (A, C, F, H, J, M, P, S, T, Y), 10 decimal digits (0–9), and 10 common objects (Apple, Car, Dog, Gold, Mobile, Rose, Scooter, Tiger, Wallet, Watch).

**Fig. 2:**
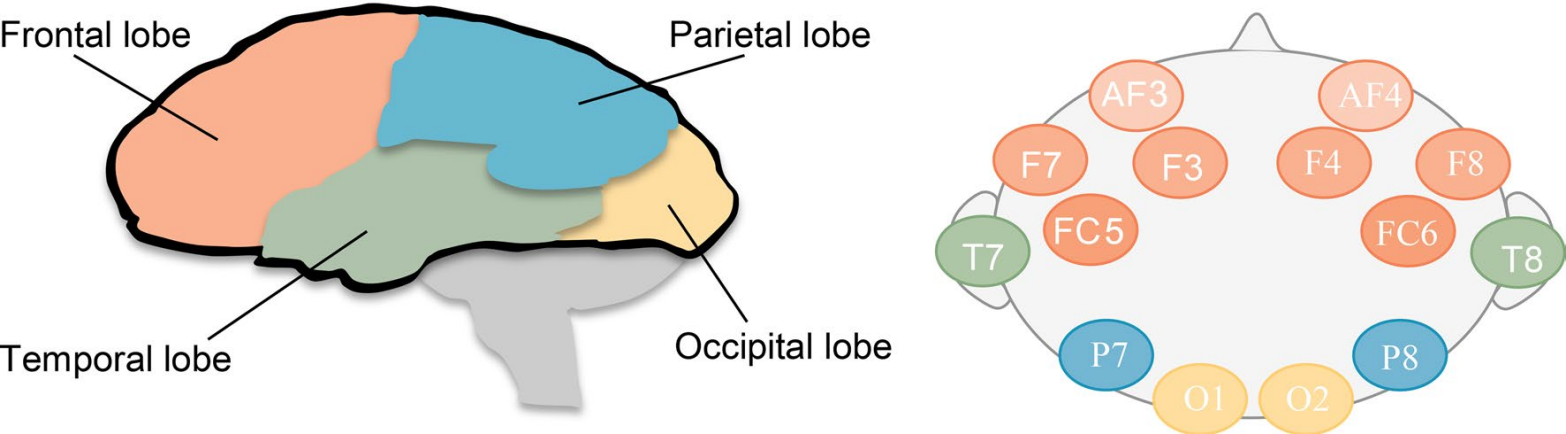
The 14-channel electrode positions on the scalp in the 10/20 international system for electroencephalogram (EEG) recordings.

EEG signals are acquired using the Emotive EPOC + 14-channel wireless headset with electrodes positioned according to the International 10–20 system at AF3, AF4, F3, F4, F7, F8, FC5, FC6, T7, T8, P7, P8, O1, and O2 as shown in Fig. 2. Signals are sampled at 128 Hz with participants imagining each class for 10 s, yielding 1,280 samples per trial.

The dataset comprises EEG recordings from 25 participants; however, data availability varies across categories. The Characters subset includes recordings from 23 participants (excluding subject IDs 23 and 24), the Digits subset includes 23 participants (excluding subject IDs 2 and 18), and the Objects subset includes 23 participants (excluding subject IDs 23 and 24). In addition, subject ID 15 contains recordings for only 29 classes and is therefore excluded. Consequently, 20 participants have complete recordings across all 30 classes and are used in experiments requiring full-category coverage. The participant selection criteria for each evaluation strategy are described in Sect.  3.4.

Five classification settings of increasing complexity are considered, based on the number and type of target classes: (1) 10-class Characters, (2) 10-class Digits, (3) 10-class Objects, (4) 20-class CharDig (characters and digits), and (5) 30-class CharDigObj (all categories). Specifically, the first three settings involve ten-class single category classification. The fourth setting considers a twenty-class classification obtained by combining two categories’ attributes. Finally, the fifth setting involves a thirty-class classification, where all categories’ attributes are jointly considered. This hierarchical structure enables analysis of model scalability across vocabulary sizes and semantic domains.

### Proposed preprocessing pipeline

EEG signals acquired during imagined speech are severely contaminated by physiological artifacts—including electrooculographic (EOG) signals from eye movements and blinks, electromyographic (EMG) signals from muscle activity, and cardiac artifacts—which overlap with neural signals predominantly. Environmental noise from participant movement and external interference further degrades signal quality. To address these challenges, an ICA-Assisted Frequency-Domain Filtering (FD-F) preprocessing framework is proposed. The method consists of a six-phase pipeline that integrates Independent Component Analysis (ICA) with frequency-domain artifact removal to maximize the signal-to-noise ratio while preserving discriminative neural patterns. The proposed preprocessing pipeline is detailed in Algorithm 1, comprises six sequential phases:

Phase 1: Independent Component Analysis (ICA) Artifact Removal: Artifact Removal is applied to raw EEG signals to decompose multi-channel data into statistically independent components, enabling separation of neural signals from artifact sources. Picard (Preconditioned ICA for Relaxed Distributions) is applied - a fast optimization method for Independent Component Analysis to decompose multi-channel data into independent components, automatically removing artifacts exceeding 2.5σ threshold while retaining 99.99% variance.

Phase 2: Frequency-Domain Transformation: In this step, Fast Fourier Transform (FFT) transforms cleaned EEG data into the frequency domain, enabling precise frequency-selective filtering with minimal phase distortion.

Phase 3: Filtering with Zero-Phase Correction: EEG signals is filtered using a zero-phase band-reject approach, suppressing the 4–15 Hz band with 2 Hz cosine-ramped transition zones to reduce spectral leakage and ringing artifacts. To preserve temporal integrity, filtering is applied in a forward–backward manner, effectively canceling phase distortions while maintaining accurate signal timing.

Phase 4: Time Domain Transformation: Inverse Fast Fourier Transform (IFFT) reconstructs filtered signals to time domain, preserving temporal patterns and dependencies essential for sequential modeling.

Phase 5: Temporal Segmentation: Filtered EEG signals are segmented using a sliding-window with two.

configurations: *Scheme A (overlapping windows)*, employing a window size of 32 samples (250 ms) and a stride of 8 to maintain consistency with prior work^24^, and *Scheme B (non-overlapping windows)*, using a window size and stride of 8 samples (62.5 ms) to prevent temporal leakage during cross-subject validation. The total number of windows generated under each scheme is given by

1 $${N_{windows}}=~{N_{recording}} \times ~\left( {\frac{{{N_{spr}} - WS}}{{Stride}}+1} \right)$$

Where ${N_{windows}}$ represents the total number of windows generated, ${N_{recording}}$ is the number of recordings, ${N_{spr}}$ is.

the number of *samples per recording* before windowing (1,280 samples for 10-second epochs at 128 Hz), *WS* is the window size (32 samples for *Scheme A*, 8 samples for *Scheme B*), and *Stride* is the step size between consecutive windows (8 samples for both schemes).

Phase 6: Adaptive Normalization: EEG signals are standardized using z-score normalization applied channel-wise. The mean and standard deviation are computed exclusively from the training data and subsequently used to normalize both training and testing samples, ensuring consistent feature space and preventing data leakage. Each signal is transformed using:

2 $${X_{norm}}=~\left( {\frac{{X - ~{\mu _{train}}}}{{{\sigma _{train}}}}} \right)$$

Where ${{\mathrm{\boldsymbol{\upmu}}}_{{\mathrm{train}}}}$ is the mean and ${{\mathrm{\boldsymbol{\upsigma}}}_{{\mathrm{train}}}}$ is the standard deviation, both computed on training data.

### Classification architectures

**Fig. 3:**
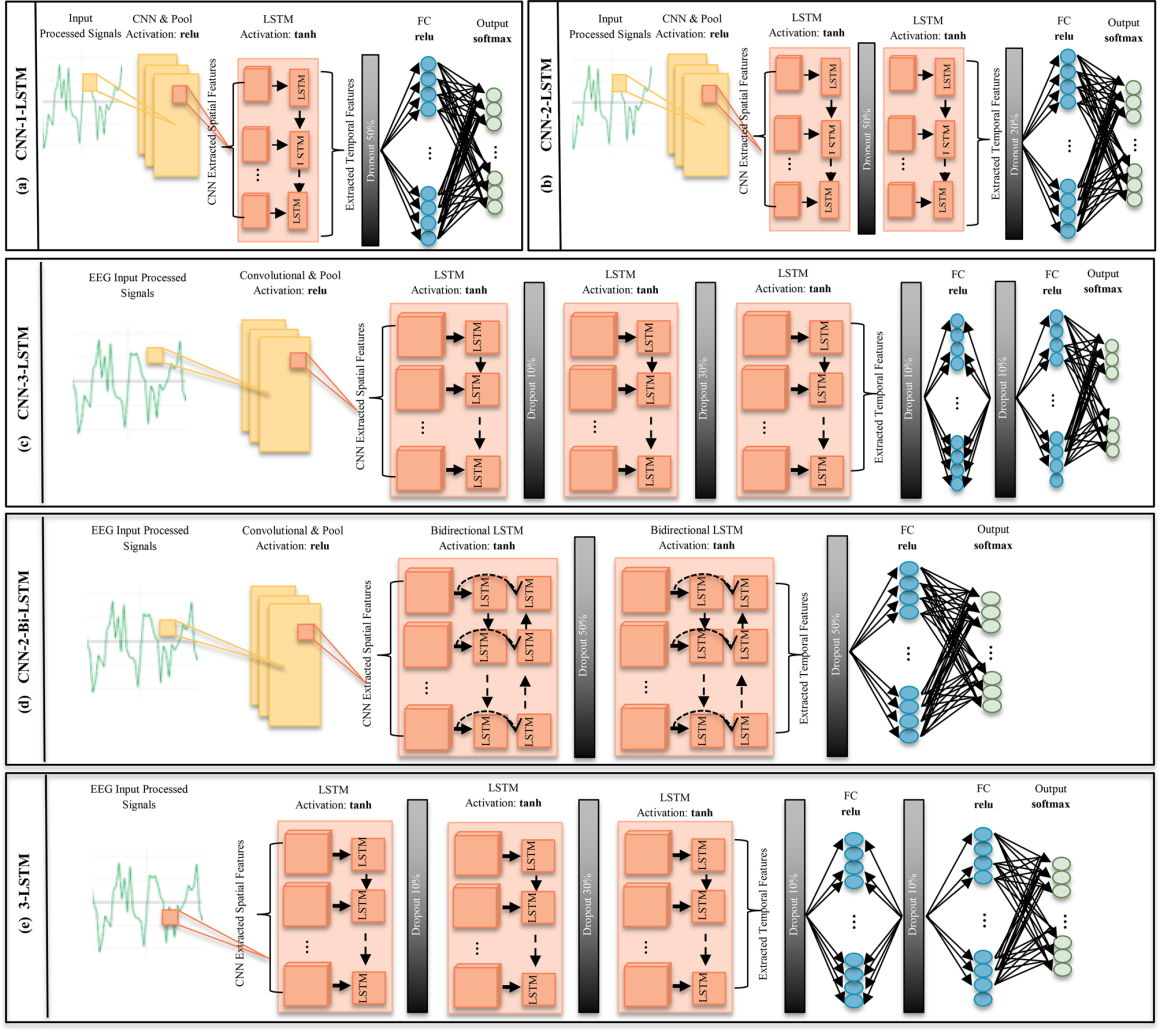
The Five proposed architectures (**a**) CNN-1-LSTM, (**b**) CNN-2-LSTM, (**c**) CNN-3-LSTM, (**d**) CNN-2-Bi-LSTM, (**e**) 3-LSTM.

To evaluate the impact of architectural depth and temporal modeling strategies on imagined speech EEG classification, five deep learning architectures are designed utilizing convolutional neural networks (CNNs), long short-term memory networks (LSTMs), and hybrid model combining both architectures as shown in Fig. 3. These models differ in their use of convolutional feature extraction, unidirectional versus bidirectional recurrent layers, and temporal depth. Among the five evaluated architectures, the simplest configuration consisting only of the one unidirectional LSTM network is treated as an internal baseline. The remaining architectures incrementally incorporate the proposed components to assess their individual and combined contributions. A concise overview of the architectural components and regularization techniques for each model is provided in Table 1. The effectiveness of the proposed approaches is subsequently evaluated through a comprehensive experimental analysis.

### Experimental setup

#### Environment and setup implementation

All experiments are conducted on Google Collaboratory (Colab Pro), a cloud-based platform that provides a Jupyter notebook environment with on-demand access to computational resources for machine learning experimentation- utilizing NVIDIA GPUs including L4, T4 High-RAM, and A100 High-RAM configurations with varying availability across experimental runs. All GPUs provided sufficient memory (> 16GB VRAM) for the models training and evaluation. System RAM is 16–24 GB depending on the allocated configuration.

**Algorithm 1:**
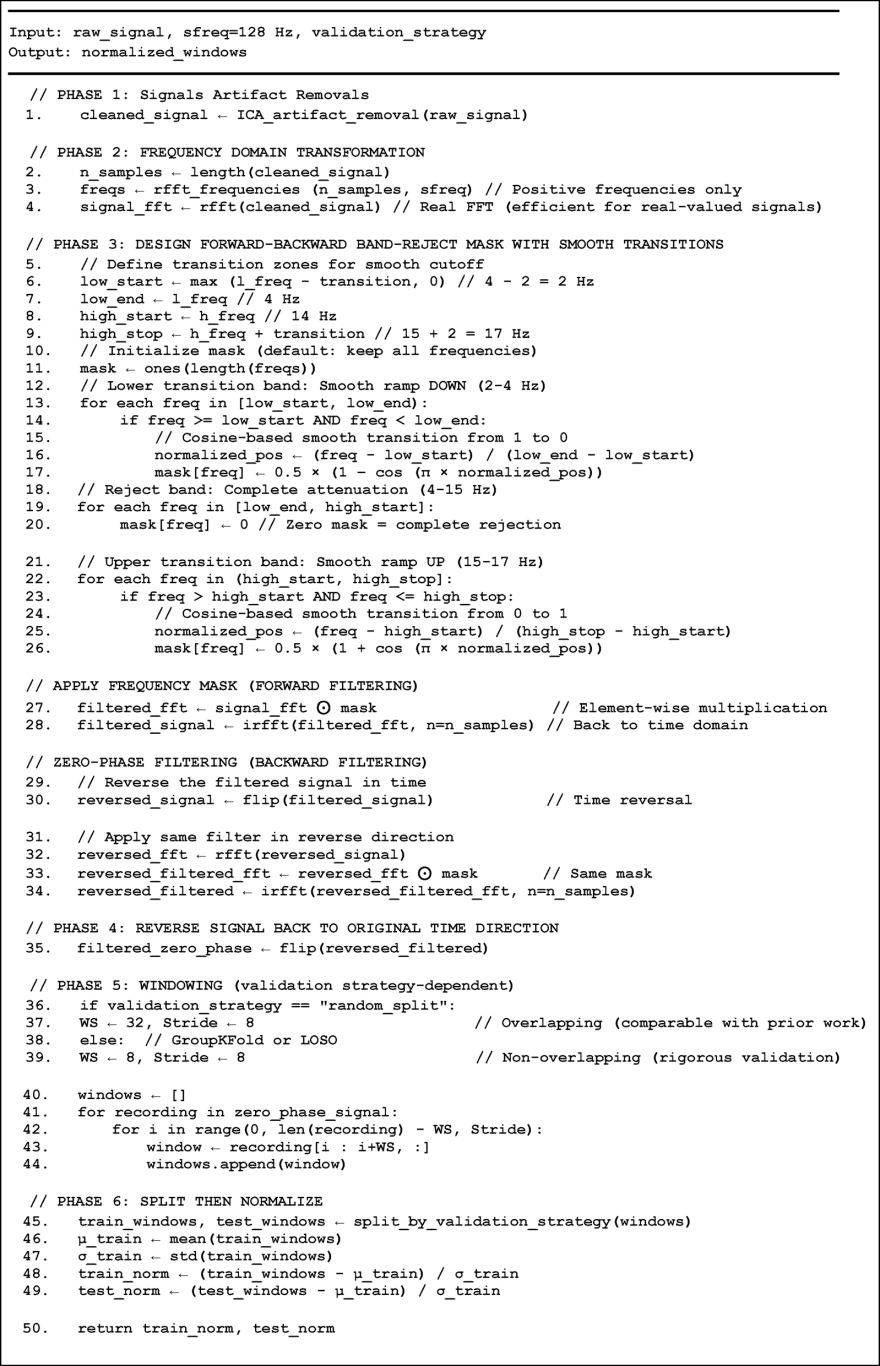
Proposed pre-processing pipeline.

The proposed CNN-LSTM architectures are implemented using TensorFlow/Keras with Python 3.10. All experiments are conducted using the stable releases of the frameworks available at the time of experimentation. Key dependencies included NumPy 1.24.3 for numerical computing, SciPy 1.10.1 for signal processing and statistical analysis, scikit-learn 1.3.0 for machine learning utilities and evaluation metrics, Pandas 2.0.3 for data manipulation, and MNE-Python 1.4.2 for baseline EEG signals preprocessing also with the Picard algorithm for EEG artifact removal. Data visualization and result presentation utilized Matplotlib 3.7.1 for general plotting, Seaborn 0.12.2 for statistical graphics and heatmaps, and standard Python libraries for figure generation and formatting.

**Table 1:** Overview of the proposed deep learning architectures for imagined speech EEG classification.

Architecture	Convolutional layers	Recurrent layers	Temporal modeling strategy	Fully connected layers	Regularization
CNN-1-LSTM (internal baseline)	Single Conv1D layer with ReLU activation, followed by batch normalization and max pooling	Single LSTM layer	Unidirectional temporal modeling	One dense layer with ReLU activation	Dropout (20%) before output
CNN-2-LSTM	Same as CNN-1-LSTM	Two stacked LSTM layers	Enhanced temporal feature learning	Same as CNN-1-LSTM	Dropout (50%) between LSTMs, 20% before output
CNN-2-Bi-LSTM	Same as CNN-1-LSTM	Two bidirectional LSTM layers	Forward–backward temporal dependency modeling	Same as CNN-1-LSTM	Dropout (50%) between LSTMs, 50% before output
CNN-3-LSTM	Same as CNN-1-LSTM	Three stacked LSTM layers	Deep temporal representation	Two dense layers	Dropout (10%, 30%, 10%) between LSTMs and 10% before output
3-LSTM	None	Three stacked LSTM layers	Pure temporal modeling	Same as CNN-1-LSTM	Same as CNN-3-LSTM

#### Training configuration

All models are trained using the Adam optimizer with learning rate α = 0.001 (β₁ = 0.9, β₂ = 0.999) and categorical cross-entropy loss. Training proceeded for maximum 500 epochs with early stopping (patience = 30, monitoring validation loss) and adaptive learning rate reduction via ReduceLROnPlateau (factor = 0.1, patience = 10). Batch sizes are 256 for standard training and 128 for LOSO calibration to accommodate smaller calibration datasets while maintaining stable gradients.

### Cross-validation strategies

Three validation strategies are implemented systematically to evaluate model generalization.

#### Strategy 1: Random split with multi-seed validation

To establish baseline performance and evaluate robustness, each of the four deep learning architectures combined with different preprocessing strategies is assessed across five independent experiments. For each experiment, the dataset is randomly divided into 90% training and 10% testing sets using stratified sampling, with different random seeds {1,2,3,4,5} used to vary the data partitioning and stochastic training processes. This strategy employs windowing *Scheme A*, which uses overlapping windows to match recent work^24^. This scheme yields a 250 ms window duration. Results are reported as mean ± margin of error (95% Confidence Interval CI) across five seeds.

The experimental results are detailed in Sect.  4.1.

#### Strategy 2: GroupKFold cross-validation

To isolate the impact of temporal dependency, ten-fold cross-validation is performed using sample-level grouping. In this study, each data sample corresponds to a fixed-length EEG window composed of multiple preprocessed signal samples; accordingly, cross-validation is conducted in a window-wise manner rather than at the preprocessed-signal level. The dataset is partitioned into ten stratified folds (k = 10), with each fold used once as the test set while the remaining nine folds form the training set. This evaluation strategy employs windowing *Scheme B*, which uses non-overlapping windows. Window-wise cross-validation strategy ensures that entire EEG windows are assigned exclusively to either training or testing folds, while non-overlapping windowing prevents shared temporal samples across windows. Together, these mechanisms eliminate temporal leakage by ensuring that no preprocessed EEG samples are shared between folds. However, subject leakage persists as subjects distribute across all folds, enabling models to learn and exploit subject-specific characteristics (brain anatomy, electrode impedance, baseline activity).

The experimental results are described in Sect.  4.2.

#### Strategy 3: Leave-one-subject-out (LOSO) cross-validation with calibration

A third subject-wise cross-validation strategy is conducted for stringent evaluation. In this strategy, one subject is held out for testing while the remaining subjects (19 subjects) are used for training, repeated for all participants. For each held-out subject, a calibration phase is performed using 20% of the subject’s data, while the remaining 80% is reserved exclusively for testing. The calibration subset is stratified to ensure equal class representation and is used to fine-tune the pretrained model, with the learning rate reset to 0.001, batch size 128, and the same optimization and early stopping criteria applied. Windowing *Scheme B* is also adopted in this strategy to prevent temporal leakage.

The experimental results are shown in Sect.  4.3.

### Evaluation metrics

Model performance is assessed by using multiple complementary metrics to ensure comprehensive evaluation across all classes. Specifically, accuracy reflects overall classification performance, balanced accuracy accounts for class imbalance, macro F1-score evaluates balanced performance across classes, per-class recall provides class-specific performance insights, and confusion matrices are used to analyze misclassification patterns. The formal definitions of each evaluation metric are presented below:

*Confusion matrix*: is a standard metric for evaluating the performance of a classification model. It summarizes the relationship between the true class labels and the predicted class labels for each class and consists of four fundamental components.

- True Positives (TP): samples correctly predicted as belonging to a given class.
- True Negatives (TN): samples correctly predicted as not belonging to that class.
- False Positives (FP): samples incorrectly predicted as belonging to that class (Type I error).
- False Negatives (FN): samples incorrectly predicted as not belonging to that class (Type II error).

*Accuracy*: measures the overall proportion of correctly classified samples across all classes.

3 $$Accuracy~=\frac{{\mathop \sum \nolimits_{{i=1}}^{C} T{P_i}}}{N}~~~~~~~~~~~$$

where C is the number of classes, $T{P_i}$ is the number of true positives for class i and N is the total number of samples.

*Precision*: measures of how many of the predicted positives are truly positive. Weighted precision aggregates per-class precision weighted by class support.

4 $$Precisio{n_{weighted}}=\mathop \sum \limits_{{i=1}}^{C} {w_i}~ \times ~Precisio{n_i}{\mathrm{where~}}Precisio{n_i}=~\frac{{T{P_i}}}{{T{P_i}~+~F{P_i}}}~$$

Where ${w_i}$ is the proportion (weight) of class i in the dataset which equals *n*_*i*_/*N* where ${n_i}$ is number of true samples in class i (support) and N is total number of samples across all classes.

Per-class recall: measures the ability of a model to correctly identify positive samples. Recall for class *i* is calculated as.

5 $$Recal{l_i}=~\frac{{T{P_i}}}{{T{P_i}~+~F{N_i}}}$$

*F1-Score*: is the harmonic mean of precision and recall, balancing false positives and false negatives. Weighted F1-Score aggregates per-class F1-scores weighted by class support.

6 $$F{1_{weighted}}=\mathop \sum \limits_{{i=1}}^{C} {w_i}~ \times ~F{1_i}{\mathrm{~where~}}F{1_i}=~2~\cdot ~\frac{{Precisio{n_i}{\mathrm{~}} \cdot Recal{l_i}}}{{~Precisio{n_i}+~Recal{l_i}}}$$

Where ${w_i}~$is the proportion (weight) of class i in the dataset which equals *n*_*i*_/*N* where ${n_i}$ is number of true samples in class i (support) and N is total number of samples across all classes.

*Macro F1-Score*: The unweighted arithmetic mean of per-class F1-scores, treating all classes equally.

7 $$F{1_{macro}}=\frac{1}{{\mathrm{C}}}~\mathop \sum \limits_{{i=1}}^{C} F{1_i}$$

where C is the number of classes.

*Balanced accuracy*: The average recall across all classes, providing equal weight to each class regardless of sample size. This metric is particularly important for assessing performance when class distributions may vary.

8 $$Balanced~Accuracy=\frac{1}{{\mathrm{C}}}~\mathop \sum \limits_{{i=1}}^{C} Recal{l_i}$$

## Results and discussion

In this section, the results of the proposed study are presented. The performance of the proposed approach is first evaluated using a random split with multi-seed validation (Strategy 1), and its results are presented in Sect. 4.1. This is followed by an evaluation using GroupKFold and Leave-One-Subject-Out (LOSO) cross-validation (Strategies 2 and 3), and their results are presented in Sect. 4.2 and 4.3, respectively. A detailed comparison with external state-of-the-art is presented. Specifically, NetTraST baseline, proposed by Ignazio et al.^24^, is evaluated under the three cross-validation strategies adopted in this study. NetTraST is a CNN–Transformer-based framework for imagined speech classification and has demonstrated strong performance on the Kumar dataset across character, digit, and object categories. In addition, a summarized comparison between the proposed approach and three other baseline methods^26,38^, and^39^ is provided. This comparison is conducted using only the random split evaluation strategy on the Kumar imagined speech dataset, ensuring a fair and consistent assessment.

### Performance of varying preprocessing methods for different architectures using random split validation strategy

This section presents the results of five proposed deep learning architectures (CNN-1-LSTM, CNN-2-LSTM, CNN-2-Bi-LSTM, CNN-3-LSTM, 3-LSTM), evaluated under different preprocessing methods and across five independent random split multi-seed experiments, as described in Sect.  3.1. Extensive quantitative results, reported through multiple tables and figures, provide a detailed comparison among the proposed architectures and specifically the enhancements against the internal baseline architecture (CNN-1-LSTM).

Initially, the five architectures are assessed under three preprocessing settings, which are defined below:

i. *Full-Band*: A no-preprocessing setting. This method utilizes the entire frequency range, applying only sliding window *Scheme A.*
ii. TD-F: The preprocessing method adopted from prior work^18–24^. A time-domain-based filter that rejects Theta (θ) and Alpha (α) bands, followed by windowing *Scheme A*.
  These two methods are intended to be used to enable a fair and controlled comparison focused solely on architectural differences.
iii. FD-F: The proposed preprocessing method in this study. An ICA-Assisted Frequency-Domain Filtering six-phase pipeline.

The resulting performance differences are analyzed to quantify the contribution of each preprocessing configuration.

Subsequently, based on the comparative analysis, the best-performing proposed architecture is selected and further evaluated using the proposed preprocessing against the baseline^24^ which is state-of-the-art framework, demonstrating its competitiveness under more challenging and realistic evaluation settings.

Tables 2 and 3 illustrate the accuracy and precision of the five proposed architectures across single categories (chars, digits, and objects) and the hybrid categories (20-class CharDig and 30-class CharDigObj) respectively using the two preprocessing methods: Full-band and TD-F. The tables further report the performance improvements achieved by each proposed architecture relative to the internal baseline (CNN-1-LSTM).

As can be analyzed from Table 2, TD-F consistently improves classification performance compared to full-band inputs for most architectures, confirming the benefit of temporal filtering in enhancing discriminative EEG patterns. Under TD-F, the CNN-1-LSTM baseline yields lower accuracies in the 86–87% range. CNN-2-LSTM delivers the best performance across the majority of the evaluated categories, reaching 92.92% (Characters), 94.11% (Digits), and 93.78% (Objects), corresponding to improvements of up to + 7.78% over the CNN-1-LSTM baseline. It is followed by CNN-2-Bi-LSTM, which attains competitive performance with accuracies of 93.85% (Characters), 92.70% (Digits), and 92.16% (Objects). Notably, CNN-2-Bi-LSTM slightly outperforms CNN-2-LSTM in the Characters task (93.85% vs. 92.92%), which may be attributed to bidirectional temporal modeling capturing longer-range contextual dependencies that are more pronounced in character-level imagined speech. The CNN-3-LSTM architecture ranks next, providing moderate gains over the baseline but showing diminishing returns with increased depth, while the standalone 3-LSTM model consistently records the weakest performance, highlighting the importance of convolutional layers for effective spatial–temporal feature extraction.

Table 3 shows that classification of imagined speech classes in an input of hybrid categories becomes increasingly challenging as the number of classes grows, with the CNN-1-LSTM baseline achieving the lowest performance in both the 20-class and 30-class tasks. In contrast, all deeper CNN–LSTM architectures benefit substantially from time-domain filtering (TD-F), yielding accuracy and precision improvements exceeding + 12% and reaching up to + 17.97% in the 30-class setting. Under TD-F, CNN-2-Bi-LSTM achieves the highest accuracy, attaining 92.65% for the 20-class task and 92.96% for the 30-class task, followed closely by CNN-3-LSTM and CNN-2-LSTM, while the standalone 3-LSTM consistently ranks below CNN-based hybrids. These results indicate that bidirectional temporal modeling combined with convolutional feature extraction is particularly effective for handling increased class diversity in hybrid imagined speech classification tasks. While accuracy and precision provide useful overall performance indicators, they may obscure class-wise behavior in multi-class settings, particularly as the number of categories increases. To ensure a class-robust and statistically reliable evaluation, the analysis in the next tables is extended to include balanced accuracy and macro-averaged F1-score, which equally weigh all classes and are less sensitive to class frequency effects. These metrics are reported under multi-seed validation to quantify both performance and uncertainty.

**Table 2:** Results for five proposed architectures across single categories using two preprocessing methods: Full-band and TD-F under random split evaluation strategy.

Architecture		Category					
		Characters			Digits		
		Full-band	TD-F	vs. Baseline (CNN-1-LSTM)	Full-bands	TD-F	vs. Baseline (CNN-1-LSTM)
CNN-1-LSTM (baseline)	Accuracy(%)	87.00 ± 0.40	87.00 ± 0.40	–	87.00 ± 0.40	87.65 ± 0.40	–
	Precision(%)	87.00 ± 0.40	87.82 ± 0.40	–	87.00 ± 0.40	87.88 ± 0.40	–
CNN-2-LSTM	Accuracy(%)	92.63 ± 0.26	92.92 ± 0.26	+ 5.92	93.16 ± 0.26	94.11 ± 0.26	+ 6.46
	Precision(%)	92.65 ± 0.26	92.97 ± 0.26	+ 5.15	93.19 ± 0.26	94.14 ± 0.26	+ 6.26
CNN-2-Bi-LSTM	Accuracy(%)	92.11 ± 0.22	93.85 ± 0.22	+ 6.85	92.92 ± 0.22	92.70 ± 0.22	+ 5.92
	Precision(%)	92.17 ± 0.22	93.89 ± 0.22	+ 6.07	92.97 ± 0.22	92.74 ± 0.22	+ 5.97
CNN-3-LSTM	Accuracy(%)	91.43 ± 0.28	93.16 ± 0.28	+ 6.16	91.97 ± 0.28	93.09 ± 0.28	+ 5.44
	Precision(%)	91.50 ± 0.28	93.16 ± 0.28	+ 5.34	92.03 ± 0.28	93.14 ± 0.28	+ 5.25
3-LSTM	Accuracy(%)	88.14 ± 0.39	90.85 ± 0.39	+ 3.85	88.81 ± 0.39	92.32 ± 0.39	+ 4.67
	Precision(%)	88.18 ± 0.39	90.91 ± 0.39	+ 3.09	88.87 ± 0.39	92.39 ± 0.39	+ 4.51

		Objects					
		Full-bands	TD-F	vs. Baseline (CNN-1-LSTM)			
CNN-1-LSTM (baseline)	Accuracy(%)	86.00 ± 0.40	86.00 ± 0.40	–			
	Precision(%)	86.19 ± 0.40	86.00 ± 0.40	–			
CNN-2-LSTM	Accuracy(%)	91.65 ± 0.26	93.78 ± 0.26	+ 7.78			
	Precision(%)	91.67 ± 0.26	93.82 ± 0.26	+ 7.82			
CNN-2-Bi-LSTM	Accuracy(%)	93.27 ± 0.22	92.16 ± 0.22	+ 7.27			
	Precision(%)	93.30 ± 0.22	92.19 ± 0.22	+ 7.11			
CNN-3-LSTM	Accuracy(%)	90.31 ± 0.28	90.38 ± 0.28	+ 4.38			
	Precision(%)	90.35 ± 0.28	90.44 ± 0.28	+ 4.44			
3-LSTM	Accuracy(%)	88.56 ± 0.39	89.77 ± 0.39	+ 3.77			
	Precision(%)	88.59 ± 0.39	89.77 ± 0.39	+ 3.77			

**Table 3:** Results for five proposed architectures across hybrid categories using two preprocessing methods: Full-band and TD-F under random split evaluation strategy.

Architecture		Category					
		20-Class (CharDig)			30-Class (CharDigObj)		
		Full-band	TD-F	vs. baseline (CNN-1-LSTM)	Full-bands	TD-F	vs. Baseline (CNN-1-LSTM)
CNN-1-LSTM (Baseline)	Accuracy(%)	80.44 ± 0.40	79.36 ± 0.40	–	76.84 ± 0.40	74.99 ± 0.40	–
	Precision(%)	80.68 ± 0.40	79.48 ± 0.40	–	77.06 ± 0.40	75.54 ± 0.40	–
CNN-2-LSTM	Accuracy(%)	91.48 ± 0.26	92.25 ± 0.26	+ 12.89	91.78 ± 0.26	91.95 ± 0.26	+ 16.96
	Precision(%)	91.58 ± 0.26	92.29 ± 0.26	+ 12.81	91.84 ± 0.26	92.02 ± 0.26	+ 16.48
CNN-2-Bi-LSTM	Accuracy(%)	91.68 ± 0.22	92.65 ± 0.22	+ 13.29	89.52 ± 0.22	92.96 ± 0.22	+ 17.97
	Precision(%)	91.72 ± 0.22	92.69 ± 0.22	+ 13.21	89.56 ± 0.22	93.00 ± 0.22	+ 17.46
CNN-3-LSTM	Accuracy(%)	90.18 ± 0.28	92.97 ± 0.28	+ 13.61	90.60 ± 0.28	91.32 ± 0.28	+ 16.33
	Precision(%)	90.27 ± 0.28	93.02 ± 0.28	+ 13.54	90.65 ± 0.28	91.40 ± 0.28	+ 15.86
3-LSTM	Accuracy(%)	89.37 ± 0.39	92.04 ± 0.39	+ 12.68	89.23 ± 0.39	90.35 ± 0.39	+ 15.36
	Precision(%)	89.44 ± 0.39	92.06 ± 0.39	+ 12.58	89.91 ± 0.39	90.43 ± 0.39	+ 14.89

Tables 4 and 5 detail the accuracy, balanced accuracy and macro-f1 of the five proposed architectures across single categories (chars, digits, and objects) and the hybrid categories (20-class CharDig and 30-class CharDigObj) respectively utilizing the proposed preprocessing method: FD-F. The tables also report the performance improvements achieved by each proposed architecture relative to the internal baseline (CNN-1-LSTM) under FD-F in addition to reporting the enhancements achieved by FD-F over the preprocessing methods: All-bands and TD-F.

**Table 4:** Results for five proposed architectures across single categories utilizing the proposed preprocessing method: FD-F under random split evaluation strategy.

Architecture		Category								
		Characters			Digits			Objects		
		FD-F	Vs Full-bands (baseline)	Vs TD-F (baseline)	FD-F	vs. Full-bands (baseline)	vs. TD-F (baseline)	FD-F	vs. Full-bands (baseline)	vs. TD-F (baseline)
CNN-1-LSTM (baseline)	Balanced Acc(%)	98.90 ± 0.48	+ 11.9	+ 11.9	98.63 ± 0.44	+ 11.62	+ 10.97	98.97 ± 0.28	+ 12.97	+ 12.97
	Accuracy (%)	98.90 ± 0.48			98.62 ± 0.44			98.97 ± 0.28		
	Macro F1(%)	98.90 ± 0.48			98.63 ± 0.44			98.97 ± 0.28		
CNN-2-LSTM	Balanced Acc(%)	99.15 ± 0.26	+ 6.51	+ 6.22	99.05 ± 0.50	+ 5.89	+ 4.94	99.21 ± 0.15	+ 5.43	+ 7.56
	Accuracy (%)	99.14 ± 0.26			99.05 ± 0.50			99.21 ± 0.15		
	Macro F1(%)	99.14 ± 0.26			99.05 ± 0.50			99.21 ± 0.15		
CNN-2-Bi-LSTM	Balanced Acc(%)	99.40 ± 0.38	+ 7.29	+ 5.55	99.17 ± 0.28	+ 6.25	+ 6.47	99.29 ± 0.28	+ 7.13	+ 6.02
	Accuracy (%)	99.40 ± 0.38			99.17 ± 0.28			99.29 ± 0.28		
	Macro F1(%)	99.40 ± 0.38			99.17 ± 0.28			99.29 ± 0.28		
CNN-3-LSTM	Balanced Acc(%)	99.19 ± 0.28	+ 7.77	+ 6.04	99.07 ± 0.26	+ 7.10	+ 5.98	99.20 ± 0.09	+ 8.82	+ 8.89
	Accuracy (%)	99.20 ± 0.28			99.07 ± 0.26			99.21 ± 0.09		
	Macro F1(%)	99.20 ± 0.28			99.07 ± 0.26			99.20 ± 0.09		
3-LSTM	Balanced Acc(%)	99.06 ± 0.42	+ 10.91	+ 8.21	98.80 ± 0.67	+ 9.99	+ 6.48	99.28 ± 0.48	+ 9.51	+ 10.72
	Accuracy (%)	99.06 ± 0.42			98.80 ± 0.67			99.28 ± 0.48		
	Macro F1(%)	99.06 ± 0.42			98.80 ± 0.67			99.28 ± 0.48		

**Table 5:** Results for five proposed architectures across hybrid categories utilizing the proposed preprocessing method: FD-F under random split evaluation strategy.

Architecture		Category							
		20-Class (CharDig)			30-Class (CharDigObj)			Training time (s)	Testing time (s)
		FD-F	Vs Full-bands (baseline)	Vs TD-F (baseline)	FD-F	vs. Full-bands (baseline)	vs. TD-F (baseline)		
CNN-1-LSTM (baseline)	Balanced Acc(%)	98.28 ± 0.45	+ 17.84	+ 18.92	97.91 ± 0.34	+ 21.06	+ 22.91	532.73 ± 88.67	1.17 ± 0.02
	Accuracy (%)	98.28 ± 0.44			97.90 ± 0.33				
	Macro F1(%)	98.28 ± 0.45			97.91 ± 0.33				
CNN-2-LSTM	Balanced Acc(%)	99.24 ± 0.26	+ 7.76	+ 6.99	99.12 ± 0.11	+ 7.34	+ 7.17	689.16 ± 44.25	1.42 ± 0.02
	Accuracy (%)	99.24 ± 0.26			99.12 ± 0.11				
	Macro F1(%)	99.24 ± 0.26			99.12 ± 0.11				
CNN-2-Bi-LSTM	Balanced Acc(%)	99.33 ± 0.08	+ 7.65	+ 6.68	99.38 ± 0.08	+ 9.86	+ 6.42	908.22 ± 131.06	2.06 ± 0.04
	Accuracy (%)	99.33 ± 0.09			99.38 ± 0.08				
	Macro F1(%)	99.33 ± 0.08			99.38 ± 0.08				
CNN-3-LSTM	Balanced Acc(%)	99.16 ± 0.38	+ 8.98	+ 6.19	99.90 ± 0.37	+ 8.50	+ 7.78	813.53 ± 62.12	1.73 ± 0.01
	Accuracy (%)	99.16 ± 0.38			99.10 ± 0.37				
	Macro F1(%)	99.16 ± 0.38			99.10 ± 0.37				
3-LSTM	Balanced Acc(%)	99.13 ± 0.24	+ 9.75	+ 7.08	99.08 ± 0.15	+ 9.86	+ 8.74	968.05 ± 248.35	1.74 ± 0.03
	Accuracy (%)	99.12 ± 0.24			99.09 ± 0.14				
	Macro F1(%)	99.13 ± 0.24			99.08 ± 0.15				

Table 4 demonstrates that the proposed FD-F preprocessing achieves superior performance over Full-Band and TD-F with increase in accuracy ranges from 4.94% to 12.97% across all the architecture. More specifically, FD-F along with the proposed CNN-2-Bi-LSTM architecture provides the highest accuracy across all task categories: 99.40% (Characters), 99.17% (Digits), and 99.29% (Objects), representing improvements of + 11.9%, + 11.62%, and + 12.97% over baseline CNN-1-LSTM, respectively. It is followed by CNN-2-LSTM, which attains balanced accuracies of 99.15%, 99.05%, and 99.21%, respectively.

The CNN-3-LSTM ranks next with slightly lower but comparable performance (99.19%, 99.07%, and 99.20%), while the standalone 3-LSTM achieves balanced accuracies of 99.06%, 98.80%, and 99.28%. The CNN-1-LSTM baseline consistently records the lowest balanced accuracy across all categories (98.90%, 98.63%, and 98.97%). The close alignment between balanced accuracy and standard accuracy metrics across all architectures indicates minimal class imbalance effects, while the consistently low variance (± 0.26–0.48 across models) demonstrates stable, reproducible performance. Comparative analysis reveals that the four proposed architectures substantially benefit from FD-F preprocessing, with bidirectional LSTM processing (CNN-2-Bi-LSTM) providing optimal spatial-temporal feature extraction for imagined speech classification across diverse semantic categories.

Table 5 presents comprehensive evaluation results for hybrid category tasks (20-class CharDig and 30-class CharDigObj) using FD-F preprocessing, demonstrating again that the CNN-2-Bi-LSTM architecture achieves state-of-the-art performance with 99.33% ± 0.08 balanced accuracy on 20-class and 99.38% ± 0.08 on 30-class tasks, representing improvements of + 7.65% and + 9.86% over baseline CNN-1-LSTM respectively. The remarkably low variance across all architectures (± 0.08–0.45) and close alignment between balanced accuracy and standard accuracy metrics confirm robust, unbiased classification performance across the expanded class set. Table 6 shows also the mean training and testing time in seconds for all of the evaluated architectures. As it can be noticed, CNN-1-LSTM achieves the lowest computational overhead, while CNN-2-LSTM and 3-LSTM incur substantially higher training costs due to increased architectural complexity. However, CNN-2-Bi-LSTM demonstrates practical deployment feasibility while achieving the highest accuracy among all evaluated architectures, thereby establishing optimal balance between performance and computational cost for hybrid-class imagined speech classification. Inference time remains consistently low across all models, with minimal variance, indicating stable and efficient deployment performance.

### Evaluation against the baseline NetTraST^24^

**Fig. 4:**
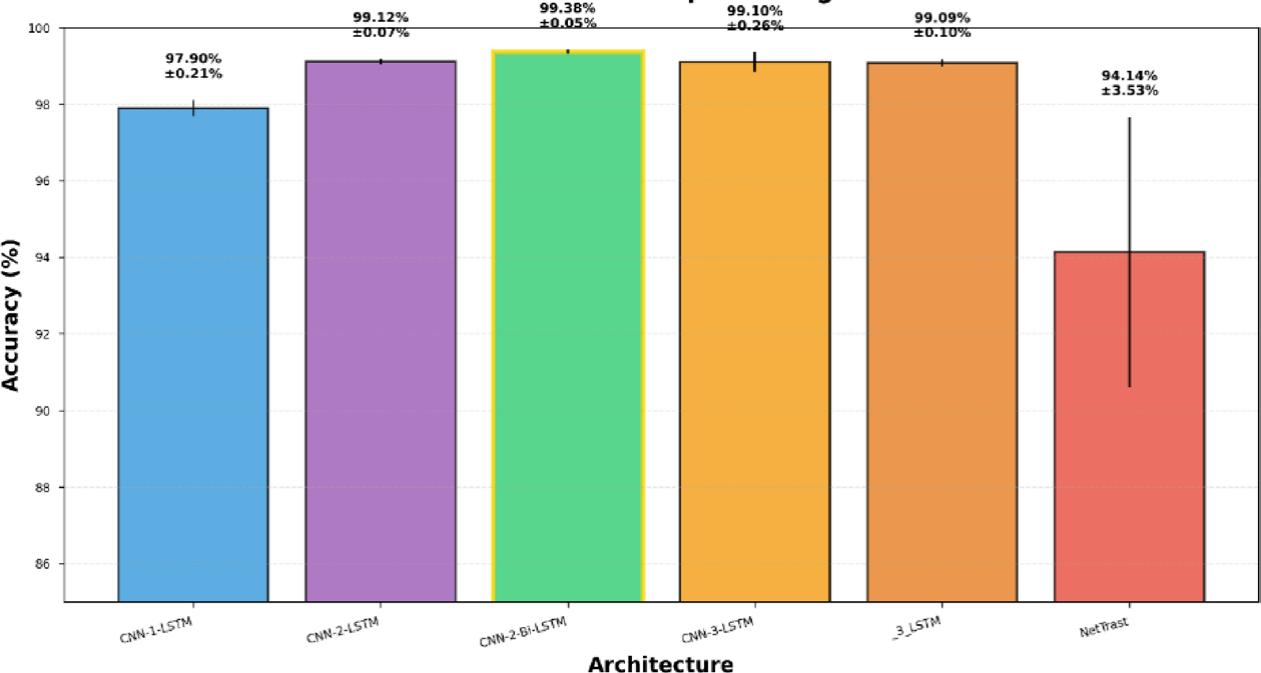
Accuracy results of the five proposed architecture utilizing FD-F against NetTraST for the 30-class CharDigObj classification under random split evaluation strategy.

Figure 4 presents a focused comparison of the evaluated five proposed architectures incorporating the proposed preprocessing method: FD-F against baseline NetTraST in terms of accuracy, for the 30-class CharDigObj classification. As it can be seen from Fig. 4, CNN-2-Bi-LSTM achieves optimal performance at 99.38% ± 0.08%, surpassing NetTraST (94.14% ± 3.53%) by + 5.24% points (t(4) = 4.12, *p* = 0.015, Cohen’s d = 2.61). Architectural progression demonstrates systematic improvement from CNN-1-LSTM baseline (97.96% ± 0.21%) to CNN-2-LSTM with additional convolutional depth (99.12% ± 0.06%, + 1.16%), culminating in CNN-2-Bi-LSTM incorporating bidirectional processing (99.38% ± 0.08%, + 0.26% from bidirectionality). Deeper variants CNN-3-LSTM (99.10%) and 3-LSTM (99.09%) achieve similar performance to CNN-2-LSTM despite increased complexity, indicating diminishing returns beyond moderate architectural depth. Figure 4 shows that all proposed architectures exhibit substantially lower variance (± 0.03% to ± 0.21%) compared to NetTraST (± 3.53%, 44-fold higher), demonstrating superior training stability. The consistent advantage of all CNN-LSTM variants over NetTraST (minimum + 3.82% points, all *p* < 0.05) establishes that architectural family contributes more substantially than specific design details, with CNN-LSTM inductive biases for spatial filtering and temporal modeling better aligned to EEG signal characteristics than transformer self-attention mechanisms.

Following the comparative analysis, the best-performing proposed architecture, CNN-2-Bi-LSTM, is selected for further evaluation. This model, combined with the proposed FD-F preprocessing, is compared against the baseline NetTraST model^24^ on the 30-class CharDigObj classification task.

**Table 6:** Results for proposed CNN-2-Bi-LSTM utilizing proposed preprocessing method FD-F versus NetTraST for the 30-class CharDigObj classification under random split evaluation strategy.

Architecture	Accuracy (%)	Balanced Acc (%)	Macro F1 (%)	Training (s)	Testing (s)
NetTraST [Ignazio 2024]	94.14 ± 3.53	94.14 ± 3.53	94.14 ± 3.53	3194.27 ± 1131.97	301.37 ± 107.79
CNN-2-Bi-LSTM	99.38 ± 0.08	99.38 ± 0.08	99.37 ± 0.08	908.22 ± 131.06	2.06 ± 0.04
Improvement (%)	+ 5.24%	+ 5.24%	+ 5.23%	3.5× faster	146× faster

Table 6 shows the accuracy, balanced accuracy and macro F1 results in addition to training and testing time for CNN-2-Bi-LSTM versus NetTraST. Table 6 also reports performance improvements achieved by CNN-2-Bi-LSTM relative to NetTraST. From Table 7, it is revealed that CNN-2-Bi-LSTM substantially outperforms the baseline NetTraST, achieving an accuracy of 99.38%±0.08 compared to 94% ± 3.53 (+ 5.24%). In addition, CNN-2-Bi-LSTM exhibits 44× lower performance variance, indicating markedly improved stability across runs. Statistical analysis confirms that this improvement is significant (t(4) = 4.12, *p* = 0.015), with a large effect size (Cohen’s d = 2.61). The magnitude of the effect size indicates that the observed performance gains are not only statistically significant but also practically meaningful.

Beyond accuracy gains, CNN-2-Bi-LSTM exhibits substantial computational advantages achieving 146× faster inference (2.06s vs. 301.37s) and 3.5× faster training (908.22s vs. 3194.27s), establishing practical feasibility for real-time BCI deployment. The combination of higher accuracy, superior stability, and substantially reduced computational cost represents an enhancement across all critical metrics, validating CNN-2-Bi-LSTM as the optimal architecture for imagined speech classification. Notably, the 2.06-second inference time (68.7ms per class) enables responsive user interaction, while NetTraST’s 301-second latency (> 5 min) renders it impractical for real-time assistive communication applications.

### GroupKFold strategy

In this section, the best-performing proposed architecture CNN-2-Bi-LSTM incorporating the proposed preprocessing strategy: FD-F is selected and further evaluated using GroupKfold cross-validation strategy described in Sect. 3.2 against the baseline NetTraST^24^ for the 30-class CharDigObj classification. GroupKFold validation serves as a critical diagnostic tool for identifying models that exploit temporal autocorrelation versus those learning genuine discriminative features. Table 8 shows that NetTraST accuracy collapses to 43.39% compared to a robust 95.25% for CNN-2-Bi-LSTMwhen temporal leakage is eliminated. The substantial performance degradation observed for NetTraST when transitioning from random split evaluation (94.14% accuracy; Table 7) to GroupKFold cross-validation (43.39% accuracy; Table 8) highlights the model’s limited generalization across subject groups. In contrast, CNN-2-Bi-LSTM maintains stable performance, with only a minor 4.13% reduction in accuracy (from 99.38% to 95.25%) which validates the robustness of the proposed approach under rigorous evaluation.

### Leave-one-subject-out (LOSO) strategy

To assess subject-independent generalization, the best-performing proposed architecture CNN-2-Bi-LSTM incorporating the proposed preprocessing strategy: FD-F is selected and evaluated using Leave-One-Subject-Out (LOSO) cross-validation strategy described in Sect. 3.3 against the baseline NetTraST^24^ for the 30-class CharDigObj classification.

Table 8 indicates that CNN-2-Bi-LSTM achieves a cross-subject classification accuracy of 78.86% ± 4.73, substantially outperforming the baseline NetTraST, which obtained 28.41% ± 4.89. The absolute improvement of 50.45% corresponds to a 177.6% relative gain, with the difference being statistically significant (t(19) = 42.64, *p* < 0.001) and associated with a large effect size (Cohen’s d = 9.54). In more details, Table 9 illustrates that CNN-2-Bi-LSTM demonstrates a moderate accuracy reduction of 20.52% when transitioning from random split evaluation (99.38%; Table 7) to LOSO cross-validation (78.86%; Table 9) compared to a pronounced accuracy drop of 65.73% (from 94.14% to 28.41%) for NetTraST clarifying limited cross-subject generalization.

**Table 7:** Results of proposed CNN-2-Bi-LSTM utilizing proposed preprocessing method FD-F versus NetTraST for the 30-class CharDigObj classification under GroupKFold cross-validation strategy.

Architecture	Accuracy (%)	Balanced Acc (%)	Macro F1(%)	Training (s)	Testing (s)
NetTraST [Ignazio 2024]	43.39 ± 1.71	43.39 ± 1.72	43.30 ± 1.50	309.23 ± 42.35	12.69 ± 1.74
CNN-2-Bi-LSTM	95.25 ± 0.52	94.14 ± 3.53	94.14 ± 3.53	904.50 ± 66.35	1.43 ± 0.03
Improvement (%)	+ 51.86	+ 50.75	+ 50.84	2.9× slower	8.9× faster

**Table 8:** Results of proposed CNN-2-Bi-LSTM utilizing proposed preprocessing method FD-F versus NetTraST for the 30-class CharDigObj classification under Leave-One-Subject-Out (LOSO) cross-validation strategy.

Architecture	Accuracy (%)	Balanced Acc (%)	Macro F1(%)	Training (s)	Testing (s)	Per-class Latency (ms/class)
NetTraST [Ignazio 2024]	28.41 ± 4.89	28.41 ± 4.89	28.25 ± 4.82	127.79 ± 22.17	4.99 ± 0.73	162.7
CNN-2-Bi-LSTM	78.86 ± 4.73	79.14 ± 4.76	78.82 ± 4.74	301.16 ± 7.16	0.77 ± 0.02	25.7
Improvement (%)	+ 50.45	+ 50.73	+ 50.57	2.4× slower	6.5× faster	6.3× faster

In addition to improved generalization, as summarized in Table 8, CNN-2-Bi-LSTM demonstrates superior computational efficiency, achieving 6.3× faster inference than NetTraST (25.7 ms vs. 162.7 ms per-class latency), thereby supporting real-time BCI operation.

#### Per-subject performance analysis

**Fig. 5:**
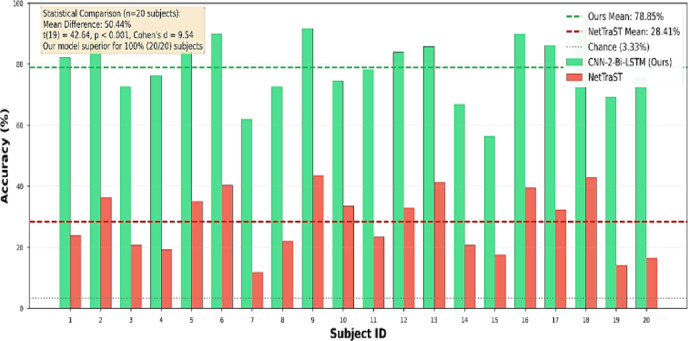
Per-subject accuracy results of proposed CNN-2-Bi-LSTM utilizing proposed preprocessing pipeline FD-F versus NetTraST for the 30-class CharDigObj classification under LOSO cross-validation strategy.

While the aggregate LOSO results provide an overall comparison between CNN-2-Bi-LSTM and NetTraST, a more detailed analysis at the subject level is necessary to assess performance consistency across individuals. To this end, Fig. 5 illustrates subject-wise accuracy results, complemented by Table 9, which reports descriptive statistics (mean, median, range, coefficient of variation (CV), standard deviation, minimum, and maximum) across subjects. Table 9 reveals remarkable consistency in CNN-2-Bi-LSTM’s superiority, outperforming NetTraST for all 20 out of 20 subjects (100% consistency, binomial test *p* < 0.001). The performance distributions are non-overlapping, as even the worst-performing subject in CNN-2-Bi-LSTM achieves 56.40% accuracy (Subject 14), exceeding NetTraST’s best subject at 43.64% (Subject 8) by + 12.76% points. This statistical guarantee of superiority, combined with 2.6× lower coefficient of variation (13.05% vs. 33.56%), establishes CNN-2-Bi-LSTM as providing both higher accuracy and more predictable outcomes across diverse individuals—critical requirements for clinical BCI deployment. Subject-level analysis shows 100% consistency (20/20 subjects favor CNN-2-Bi-LSTM).

#### Per-class performance analysis

**Fig. 6:**
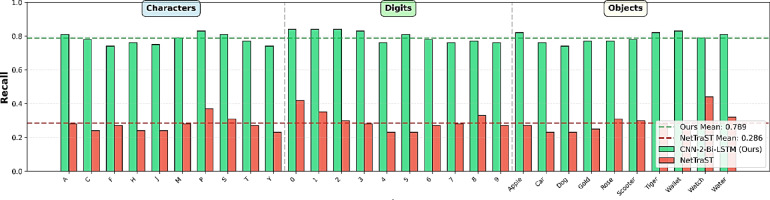
Per-class recall results of proposed CNN-2-Bi-LSTM utilizing proposed preprocessing method FD-F versus NetTraST for the 30-class CharDigObj classification under LOSO cross-validation strategy.

**Fig. 7:**
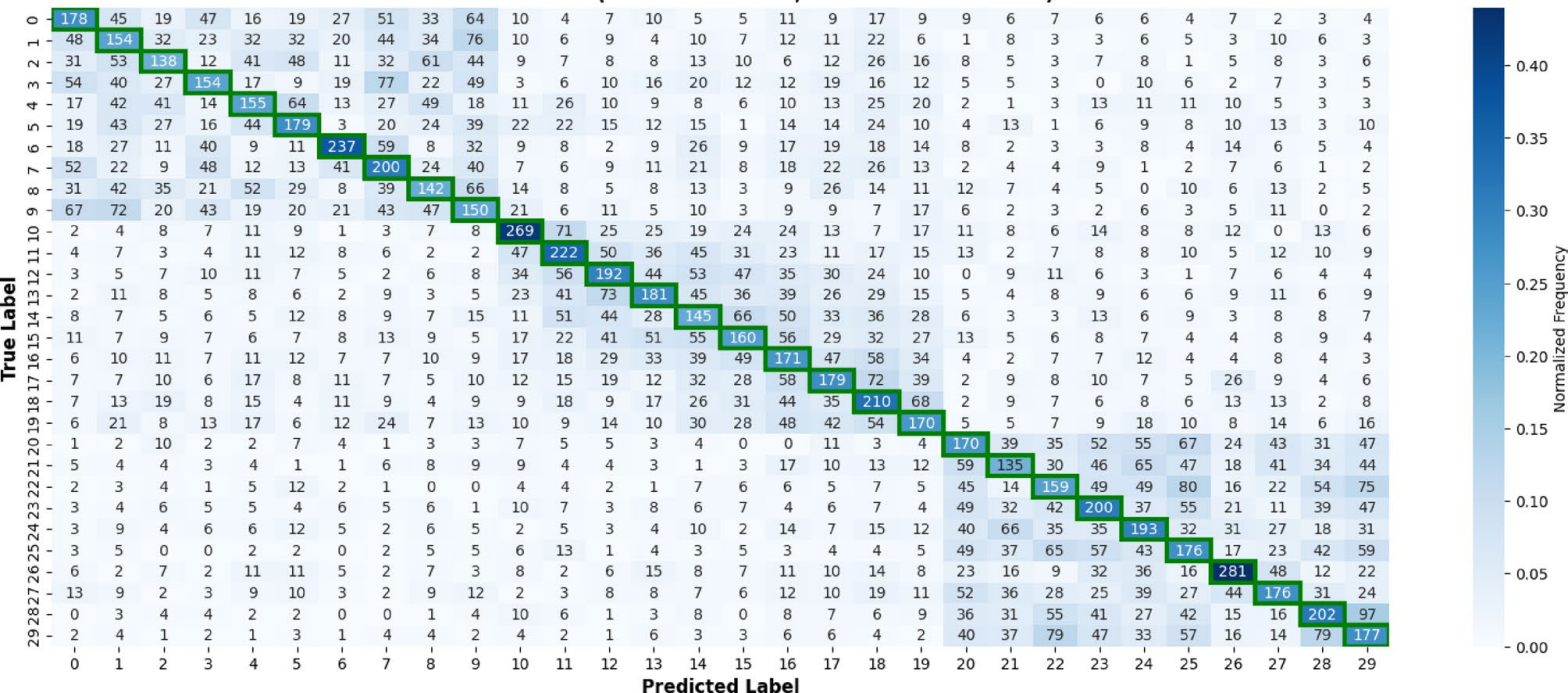
Confusion matrix results of NetTraST for the 30-class CharDigObj classification under LOSO cross-validation strategy.

**Fig. 8:**
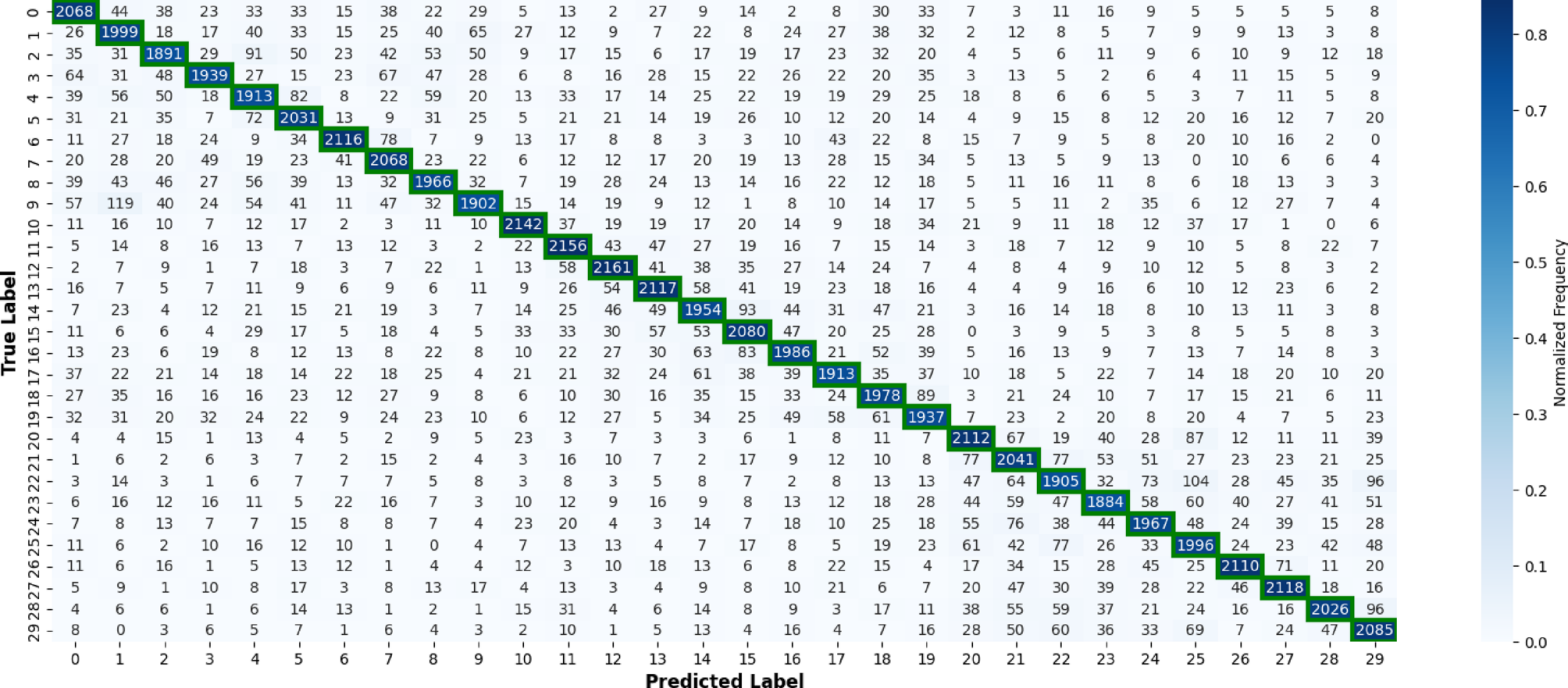
Confusion matrix results of CNN-2-Bi-LSTM utilizing proposed preprocessing method FD-F for the 30-class CharDigObj classification under LOSO cross-validation strategy.

Beyond subject-level variability, it is also important to examine how CNN-2-Bi-LSTM and NetTraST perform across different classes. Accordingly, Fig. 6 reports per-class recall across all 20 LOSO iterations, while Figs. 7 and 8 present the corresponding confusion matrices, providing deeper insight into class-specific performance and misclassification patterns. It can be inferred that CNN-2-Bi-LSTM consistently achieves substantially higher recall across all 30 classes, with no class exhibiting degraded performance relative to NetTraST. This uniform advantage is further reflected in the confusion matrix of Fig. 7, which shows a strong diagonal structure with limited off-diagonal confusion, indicating effective class separation under cross-subject evaluation. In contrast, NetTraST (Fig. 8) exhibits widespread misclassification, weaker diagonal dominance, and pronounced confusion across multiple classes, suggesting limited robustness to inter-subject variability. Together, these results confirm that the proposed model not only improves average accuracy under LOSO, but also delivers stable and reliable recognition across all semantic classes, which is essential for practical imagined speech BCI deployment.

**Table 9:** Subject Performance Distribution of proposed CNN-2-Bi-LSTM utilizing proposed preprocessing method FD-F versus NetTraST for the 30-class CharDigObj classification under LOSO cross-validation strategy.

Statistic	CNN-2-Bi-LSTM	NetTraST	Difference
Mean	78.86%	28.41%	+ 50.45%
Median	80.59%	28.17%	+ 52.42%
Min	56.40% (Subj 14)	11.87% (Subj 6)	+ 44.53%
Max	91.66% (Subj 8)	43.64% (Subj 8)	+ 48.02%
Range	35.26%	31.77%	–
Std dev	9.84%	10.18%	–
CV	13.05%	33.56%	–

**Fig. 9:**
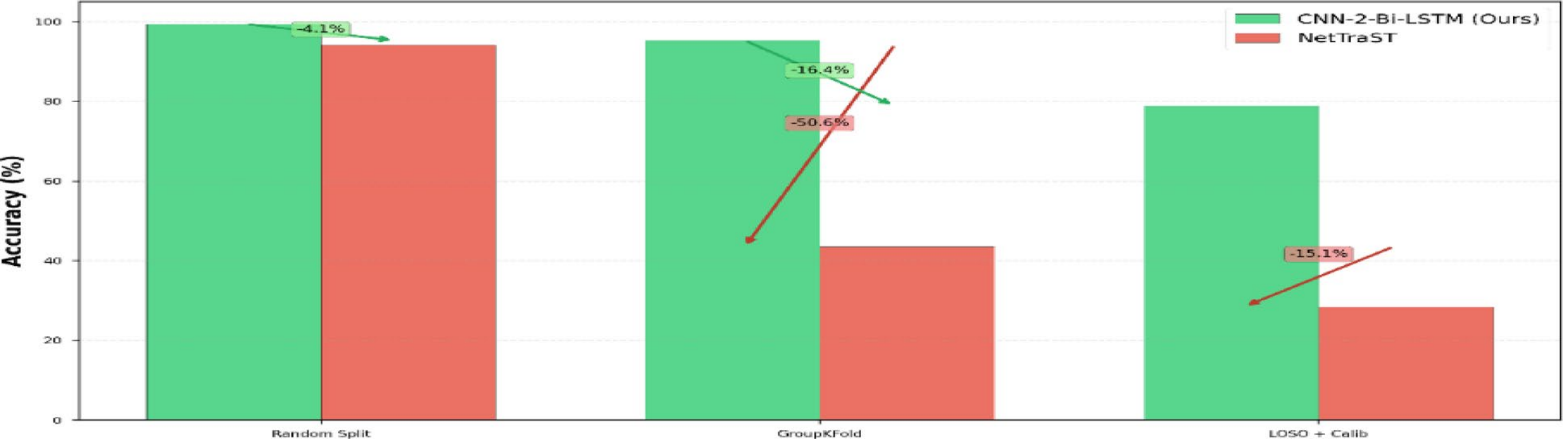
Accuracy results of CNN-2-Bi-LSTM utilizing proposed preprocessing method FD-F against the baseline NetTraST for the 30-class CharDigObj classification across the three evaluation strategies considered in this study: random split, GroupKFold, and Leave-One-Subject-Out (LOSO).

### Cross-strategy performance comparison

Finally, Fig. 9 compares the accuracy of CNN-2-Bi-LSTM utilizing proposed preprocessing method FD-F against.

the baseline NetTraST for the 30-class CharDigObj classification across the three evaluation strategies considered in this study: random split, GroupKFold, and Leave-One-Subject-Out (LOSO). Figure 9 shows that shows that while NetTraST performs competitively under random split evaluation, its accuracy decreases markedly under GroupKFold and LOSO protocols. In contrast, the proposed CNN-2-Bi-LSTM maintains consistently higher accuracy across all strategies, indicating improved robustness to inter-subject variability and more reliable generalization under subject-aware validation.

### Architectural complexity and parameter analysis

This section presents a comparative analysis of the architectural complexity of the evaluated architectures, with particular emphasis on trainable parameter counts and computational efficiency. Table 10 presents the total and trainable parameter counts, and model sizes in MB for all evaluated architectures. Model size is computed as the total number of trainable parameters multiplied by 4 bytes, assuming float32 precision. As can be seen, CNN-2-Bi-LSTM comprises of 2.45 million trainable parameters, approximately 2.4 times more than NetTraST. The increased parameter count arises primarily from the use of bidirectional LSTM layers. In contrast, the simpler CNN-1-LSTM architecture, which contains only 450 K parameters, achieves a lower accuracy of 97.90%, indicating that architectural simplicity alone is insufficient. The superior performance of CNN-2-Bi-LSTM (99.38%) demonstrates that the additional ~ 2 million parameters enable the model to capture richer temporal dependencies, which are critical for high-accuracy imagined speech classification.

**Table 10:** Comparison of architectural complexity and parameters count of the evaluated architectures.

Architecture	Total parameters	Trainable parameters	Model size (MB)
CNN-1-LSTM (Baseline)	450,646	449,850	1.72
CNN-2-LSTM	975,958	975,162	3.72
CNN-2-Bi-LSTM	2,453,590	2,452,282	9.36
CNN-3-LSTM	1,507,606	1,506,810	5.75
3-LSTM	1,372,310	1,371,770	5.23
NetTraST	1,002,088	1,002,088	3.82

### Additional state-of-the-art comparison

This section presents a summarized comparison of the proposed five architectures utilizing proposed preprocessing method FD-F against state-of-the-art studies evaluated on the Kumar imagined speech dataset across single categories under simple random split strategy. Table 11 reports the accuracy of each study demonstrating that the proposed approach achieves state-of-the-art performance across all three individual categories—Characters (99.40%), Digits (99.17%), and Objects (99.29%)—substantially outperforming previous approaches including Kumar et al.‘s^26^ Random Forest that achieved accuracies of 66.90%, 68.50%, and 65.70%, while Tirupattur et al.‘s^38^ reported 71.20%, 72.90%, and 73.00% using a CNN-based mode; A subsequent CNN-LSTM approach by Kumar et al.^39^ improved performance to 87.30%, 85.90%, and 87.50% across the same categories; More recent Ignazio et.at^24^. employed a CNN-transformer architecture (NetTraST), achieving 97.30%, 97.20%, and 96.60% for Characters, Digits, and Objects respectively. From Table 12, it is illustrated that CNN-2-Bi-LSTM establishes new performance benchmarks across all semantic categories. Moreover, Table 12 reveals consistent performance across the proposed five architectural variants (CNN-1-LSTM, CNN-2-Bi-LSTM, CNN-2-LSTM, CNN-3-LSTM, 3-LSTM) all exceeding 99% accuracy on 10-class single category classification, demonstrating that the proposed preprocessing method: FD-F enables multiple architectures to, thereby validating that the primary contribution stems from the proposed six-phase preprocessing pipeline combined with architectural design in bidirectional temporal processing.

## Conclusion

This study proposes an approach to addresses key challenges in EEG-based imagined speech classification by systematically integrating architectural design, frequency-domain preprocessing, and rigorous cross-subject validation. The approach is evaluated on a large, imagined speech vocabulary comprising 30 classes, spanning characters, digits, and objects. The results demonstrate that reliable imagined speech decoding requires not only expressive neural architectures but also effective artifact mitigation and realistic evaluation strategies that reflect practical deployment conditions. Through a structured comparison of CNN–LSTM hybrid models, bidirectional temporal modeling emerged as the most effective architectural choice, indicating that imagined speech EEG signals contain informative temporal dependencies in both forward and backward directions. In parallel, the proposed frequency-domain preprocessing pipeline—combining Independent Component Analysis with zero-phase band-reject filtering and adaptive normalization—consistently improved classification performance across architectures, highlighting the importance of advanced artifact attenuation beyond conventional temporal filtering. A central contribution of this work lies in its rigorous validation approach. By evaluating models under random splits, GroupKFold cross-validation, and Leave-One-Subject-Out (LOSO) evaluation with limited calibration, it is demonstrated that commonly used validation strategies substantially overestimate performance due to temporal and subject leakage. Under leakage-free cross-subject evaluation, the proposed approach maintained robust performance with minimal calibration, providing a more realistic estimate of achievable accuracy for unseen users. Balanced per-class performance and consistent improvements across subjects indicate that the proposed approach scales beyond small-vocabulary settings and represents a step toward functional imagined speech BCIs. Importantly, the combination of high accuracy, low variance, and efficient inference supports the feasibility of real-time implementation. In summary, this work demonstrates that imagined speech BCIs can achieve robust large-vocabulary decoding when advanced preprocessing, appropriate architectural choices, and leakage-free validation are jointly considered. The proposed methodology and evaluation approach provide a foundation for future research aimed at translating imagined speech decoding from controlled laboratory settings to real-world assistive communication systems.

**Table 11:** Comparison of the proposed approach against state-of-the-art studies evaluated on the Kumar imagined speech dataset across single categories.

Research	Architecture	Char (%)	Digit (%)	Obj (%)	Year
Kumar et al.^26^	Random Forest	66.90	68.50	65.70	2018
Tirupattur et al.^38^	CNN	71.20	72.90	73.00	2018
Ignazio et al.^24^	CNN/transformers	97.30	97.20	96.60	2024
Kumar et al.^39^	CNN/LSTM	87.30	85.90	87.50	2022
Proposed system	*CNN-2-LSTM*	99.14	99.05	99.21	2025
	*CNN-2-Bi-LSTM*	99.40	99.17	99.29	
	*CNN-3-LSTM*	99.20	99.07	99.20	
	*3-LSTM*	99.06	98.80	99.28	

## Data Availability

The Kumar EEG Imagined Speech Dataset^26^, which is raw data used in this study. The dataset is publicly available on Kaggle at the following URL: https://www.kaggle.com/datasets/ignazio/kumars-eeg-imagined-speech.
